# Supplementary material for: Measurement of Fitness and Predatory Ability of Four Predatory Mite Species in Tibetan Plateau under Laboratory Conditions
Source: Insects. 2024 Feb 6;15(2):119. doi: 10.3390/insects15020119 (PMC10889832; doi:10.3390/insects15020119)
Supplement: Supplementary file 1 [file insects-15-00119-s001.zip › insects-2794768-supplementary.pdf]

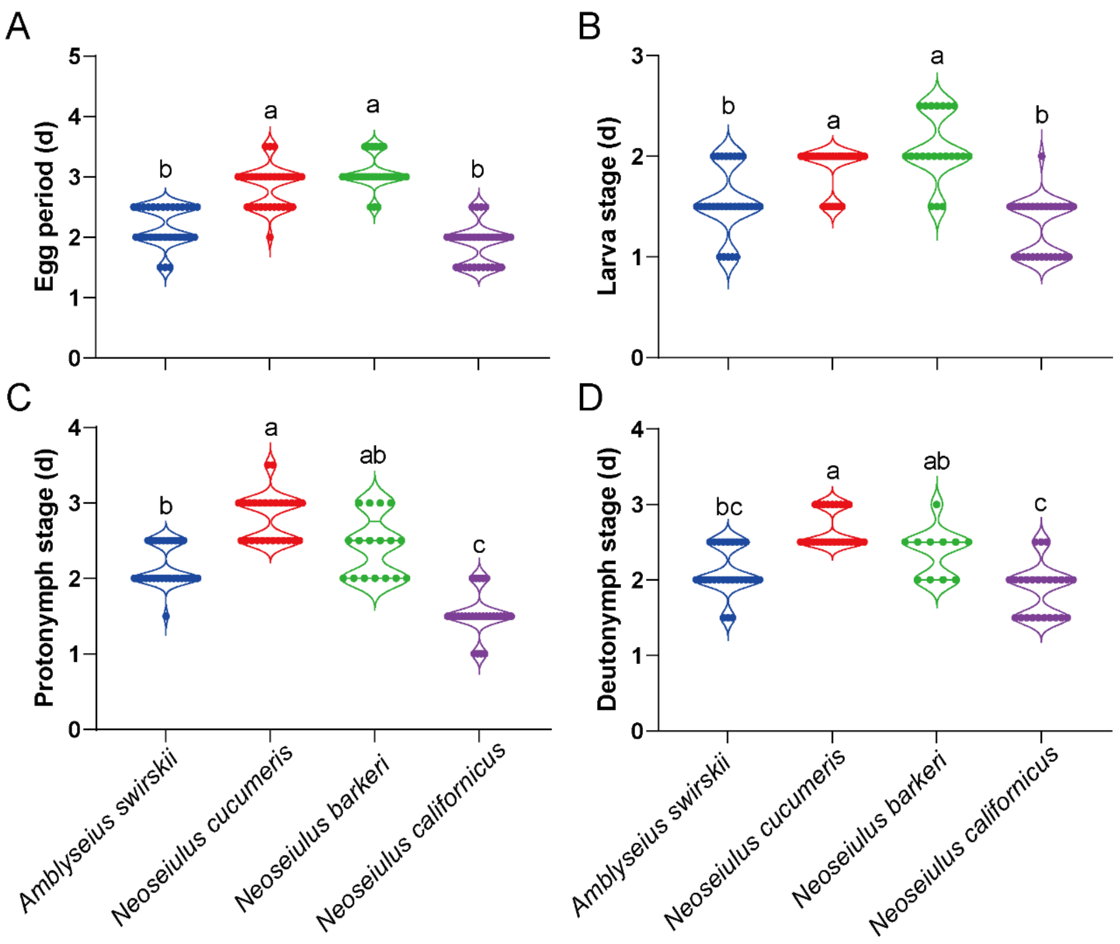

Figure S1. Developmental time of different growth stages of four predatory mite species in Tibetan Plateau, the egg period (A), larva stage (B), protonymph stage (C) and deutonymph stage (D) were listed, respectively. The same letters above columns represent homogeneous groups in post-hoc tests ($P > 0.05$) following an ANOVA.

Table S1. Parameter estimates of four predatory species' functional response when feeding on different stages of TSSM by Holling II functional response model (after release for 1d), the 1st repeat.

**Predatory mite species**	***Tetranychus urticae* (green) stage**	**Functional response model (N_a=)**	***R*2**	**Chi-square value**	***P* value**
Amblyseius swirskii	adult	$0.238N_0/(1+0.206\ N_0)$	0.992	120.000	0.000
	nymph	$1.346\ N_0/(1+0.751\ N_0)$	0.751	83.905	0.000
	egg	$1.178\ N_0/(1+0.506\ N_0)$	0.767	49.699	0.002
Neoseiulus cucumeris	adult	$0.238\ N_0/(1+0.329\ N_0)$	0.783	0.000	0.000
	nymph	$0.759\ N_0/(1+0.289\ N_0)$	0.925	50.400	0.000
	egg	$0.928\ N_0/(1+0.186\ N_0)$	0.922	71.429	0.000
Neoseiulus barkeri	adult	$0.246\ N_0/(1+0.241\ N_0)$	0.988	90.000	0.000
	nymph	$0.925\ N_0/(1+0.561\ N_0)$	0.873	40.000	0.000
	egg	$0.808\ N_0/(1+0.343\ N_0)$	0.879	40.000	0.000
Neoseiulus californicus	adult	$0.246\ N_0/(1+0.241\ N_0)$	0.985	93.333	0.000
	nymph	$0.925\ N_0/(1+0.561\ N_0)$	0.965	98.800	0.000
	egg	$0.808\ N_0/(1+0.343\ N_0)$	0.826	61.200	0.000

Table S2. Parameter estimates of four predatory species' functional response when feeding on different stages of TSSM by Holling II functional response model (after release for 1d), the 2nd repeat.

**Predatory mite species**	***Tetranychus urticae* (green) stage**	**Functional response model (N_a=)**	***R*2**	**Chi-square value**	***P* value**
Amblyseius swirskii	adult	0.204N/(1+0.048N)	0.977	84.308	0.000
	nymph	0.894N/(1+0.132N)	0.893	73.943	0.004
	egg	0.605N/(1+0.027N)	0.965	100.457	0.000
Neoseiulus cucumeris	adult	0.430N/(1+0.079N)	0.963	64.286	0.002
	nymph	0.629N/(1+0.063N)	0.963	84.600	0.000
	egg	0.835N/(1+0.043N)	0.986	72.600	0.001
Neoseiulus barkeri	adult	0.235N/(1+0.160N)	0.978	90.000	0.000
	nymph	0.453N/(1+0.117N)	0.949	44.970	0.001
	egg	0.973N/(1+0.244N)	0.875	56.800	0.002
Neoseiulus californicus	adult	0.199N/(1+0.026N)	0.973	66.458	0.000
	nymph	1.020N/(1+0.352N)	0.760	61.857	0.003
	egg	1.098N/(1+0.146N)	0.912	69.333	0.000

Table S3. Parameter estimates of four predatory species' functional response when feeding on different stages of TSSM by Holling II functional response model (after release for 1d), the 3rd repeat.

**Predatory mite species**	***Tetranychus urticae* (green) stage**	**Functional response model (N_a=)**	***R*2**	**Chi-square value**	***P* value**
Amblyseius swirskii	adult	0.431N/(1+0.064N)	0.961	49.727	0.002
	nymph	0.935N/(1+0.180N)	0.941	71.000	0.000
	egg	0.885N/(1+0.126N)	0.946	73.000	0.001
Neoseiulus cucumeris	adult	0.426N/(1+0.117N)	0.916	91.700	0.000
	nymph	0.662N/(1+0.118N)	0.907	58.800	0.001
	egg	1.028N/(1+0.112N)	0.920	69.600	0.011
Neoseiulus barkeri	adult	0.529N/(1+0.385N)	0.886	99.500	0.000
	nymph	0.703N/(1+0.172N)	0.914	54.600	0.004
	egg	0.882N/(1+0.122N)	0.940	67.500	0.001
Neoseiulus californicus	adult	0.445N/(1+0.196N)	0.919	83.786	0.000
	nymph	0.657N/(1+0.083N)	0.934	62.700	0.000
	egg	1.059N/(1+0.133N)	0.917	59.686	0.023

Table S4. Parameter estimates of four predatory species' functional response when feeding on different stages of TSSM by Holling II functional response model (after release for 15d), the 1st repeat.

**Predatory mite species**	***Tetranychus urticae* (green)**	**Functional response equation**	***R*2**	**Chi-square value**	***P* value**
Amblyseius swirskii	adult	0.209N/(1+0.075N)	0.982	104.933	0.000
	nymph	0.631N/(1+0.091N)	0.939	79.200	0.000
	egg	1.064N/(1+0.097N)	0.929	85.000	0.000
Neoseiulus cucumeris	adult	0.214N/(1+0.063N)	0.995	99.545	0.000
	nymph	0.645N/(1+0.096N)	0.915	66.800	0.001
	egg	0.867N/(1+0.075N)	0.979	77.357	0.000
Neoseiulus barkeri	adult	0.240N/(1+0.340N)	0.784	120.00	0.000
	nymph	0.466N/(1+0.234N)	0.899	109.200	0.000
	adult	0.701N/(1+0.225N)	0.914	102.600	0.000
Neoseiulus californicus	adult	0.853N/(1+0.491N)	0.844	75.900	0.000
	nymph	1.093N/(1+0.131N)	0.921	87.000	0.000
	adult	1.170N/(1+0.503N)	0.820	49.371	0.000

Table S5. Parameter estimates of four predatory species' functional response when feeding on different stages of TSSM by Holling II functional response model (after release for 15d), the 2nd repeat.

**Predatory mite species**	***Tetranychus urticae* (green)**	**Functional response equation**	***R*2**	**Chi-sqaure value**	***P* value**
Amblyseius swirskii	adult	0.459N/(1+0.018N)	0.913	66.400	0.060
	nymph	0.919N/(1+0.154N)	0.934	62.800	0.000
	egg	0.731N/(1+0.029N)	0.930	69.600	0.003
Neoseiulus cucumeris	adult	0.196N/(1+0.010N)	0.968	94.857	0.000
	nymph	1.171N/(1+0.308N)	0.821	66.229	0.000
	egg	1.099N/(1+0.100N)	0.977	92.200	0.000
Neoseiulus barkeri	adult	0.428N/(1+0.092N)	0.956	71.500	0.000
	nymph	0.663N/(1+0.105N)	0.930	53.000	0.006
	adult	0.644N/(1+0.062N)	0.939	61.257	0.017
Neoseiulus californicus	adult	0.197N/(1+0.010N)	0.963	104.000	0.000
	nymph	0.861N/(1+0.076N)	0.988	96.200	0.000
	adult	1.104N/(1+0.123N)	0.898	77.000	0.000

Table S6. Parameter estimates of four predatory species' functional response when feeding on different stages of TSSM by Holling II functional response model (after release for 15d), the 3rd repeat.

**Predatory mite species**	***Tetranychus urticae* (green)**	**Functional response equation**	***R*2**	**Chi-sqaure value**	***P* value**
Amblyseius swirskii	adult	0.940N/(1+0.195N)	0.918	65.829	0.006
	nymph	0.903N/(1+0.153N)	0.917	67.400	0.000
	egg	0.608N/(1+0.030N)	0.947	88.200	0.001
Neoseiulus cucumeris	adult	0.455N/(1+0.195N)	0.910	97.500	0.000
	nymph	1.046N/(1+0.228N)	0.852	69.700	0.002
	egg	0.833N/(1+0.054N)	0.950	69.200	0.012
Neoseiulus barkeri	adult	0.432N/(1+0.126N)	0.927	70.714	0.002
	nymph	0.657N/(1+0.112N)	0.959	63.467	0.000
	adult	0.653N/(1+0.076N)	0.907	73.600	0.001
Neoseiulus californicus	adult	0.438N/(1+0.164N)	0.913	93.000	0.000
	nymph	0.830N/(1+0.078N)	0.903	82.000	0.001
	adult	0.605N/(1+0.051N)	0.864	83.400	0.000

Table S7. Multiple linear regression analysis of the attack rate (ɑ) with predatory mite species, TSSM growth stages and predatory period.

	B	***β***	***t***	**p-value**	***f***	***R2***
predatory mite species	3.199	2.832	7.619	< 0.001	20.460	0.451
TSSM growth stages	-1.064	-2.908	-7.824	< 0.001		
predatory period	-0.006	-0.035	-0.394	0.695		
attack rate (ɑ)	0.930		2.741	0.008		

Table S8. Multiple linear regression analysis of the handling time (T_h) with predatory mite species, TSSM growth stages and predatory period.

	B	***β***	***t***	**p-value**	***f***	***R2***
predatory mite species	-7.860	-1.483	-3.278	0.002	6.443	0.221
TSSM growth stages	2.242	1.1306	2.887	0.005		
predatory period	0.240	0.283	2.645	0.010		
handling time (Th)	10.109		5.373	< 0.001		
